# Abnormal expression of mRNA, microRNA alteration and aberrant DNA methylation patterns in rectal adenocarcinoma

**DOI:** 10.1371/journal.pone.0174461

**Published:** 2017-03-28

**Authors:** Yang Hua, Xiukun Ma, Xianglong Liu, Xiangfei Yuan, Hai Qin, Xipeng Zhang

**Affiliations:** 1 Department of Colorectal Surgery, Tianjin Union Medical Center, Tianjin, China; 2 Department of Surgery, Sino-Singapore Eco-City Hospital of Tianjin Medical University, Tianjin, China; 3 Tianjin Institute of Integrative Medicine for Acute Abdominal Diseases, Nankai Hospital, Tianjin, China; University of Navarra, SPAIN

## Abstract

**Aim:**

Rectal adenocarcinoma (READ) is a malignancy cancer with the high morbidity and motility worldwide. Our study aimed to explore the potential pathogenesis of READ through integrated analysis of gene expression profiling and DNA methylation data.

**Methods:**

The miRNA, mRNA expression profiling and corresponding DNA methylation data were downloaded from The Cancer Genome Atlas (TCGA) database. Differentially expressed mRNAs/ miRNAs/methylated regions (DEmRNA/DEmiRNAs) were identified in READ. The negatively correlation of DEmiRNA-DEmRNAs and DNA methylation-DEmRNAs were obtained. DEmRNAs expression was validated through quantitative real-time polymerase chain reaction (qRT-PCR) and microarray expression profiling analyses.

**Results:**

1192 dysregulated DEmRNAs, 27 dysregulated DEmiRNAs and 6403 aberrant methylation CpG sites were screened in READ compared to normal controls. 1987 negative interaction pairs among 27 DEmiRNAs and 668 DEmRNAs were predicted. 446 genes with aberrant methylation were annotated. Eventually, 50 DEmRNAs (39 down- and 11 up-regulated DEmRNAs) with hypermethylation, synchronously negatively targeted by DEmiRNAs, were identified through the correlation analysis among 446 genes with aberrant methylation and 668 DEmRNAs. 50 DEmRNAs were significantly enriched in cAMP signaling pathway, circadian entrainment and glutamatergic synapse. The validation results of expression levels of DEmRNAs through qRT-PCR and microarray analyses were compatible with our study.

**Conclusion:**

7 genes of SORCS1, PDZRN4, LONRF2, CNGA3, HAND2, RSPO2 and GNAO1 with hypermethylation and negatively regulation by DEmiRNAs might contribute to the tumorigenesis of READ. Our work might provide valuable foundation for the READ in mechanism elucidation, early diagnosis and therapeutic target identification.

## Introduction

Colorectal cancer (CRC) is a leading cause of cancers with high morbidity and mortality. CRC is the third leading cause of cancer both in male and female and worldwide number of death in 2012 is more than 690,000 [1]. Australia/New Zealand, Europe and Northern America possess the highest incident rates, Africa and Northern America possess the low incidence rates of CRC [1, 2].

CRC is classified as colon cancer and rectum cancer according to the anatomical location. Development of metastasis often meddles with homeostasis and predicts unfavorable prognosis [3]. Approximately 20% of CRC patients lose opportunity for radical surgery on account of metastases [4]. Rectal cancer spreads more frequently to the thoracic organs, bone and nervous system and less frequently to peritoneum compared to colon cancer [5]. Age, gender, smoking and diabetes mellitus are risk factors of rectal cancer [6–8].

Numerous data indicate that the aberrant accumulation of genetic and epigenetic changes function as vital roles in initiation and development of rectal cancer.

DNA methylation typically occurs at cytosine-phosphate-guanine (CpG) sites, regulates gene expression, protein function and RNA processing. Patients with hypomethylation of LINE-1 show shorter survival time and a higher incidence rate of tumor recurrence in early-stage rectal cancer (stage I-II) [9]. KRAS mutations and CDKN2A promoter methylation are closely related to the poor overall survival in rectal cancer [10]. XRCC3 is over-expressed in rectal cancer patients responding to preoperative chemoradiotherapy (pCRT) followed by surgery compared with those non-responders, the deregulation of which is extensively involved in the chemo-resistance mechanism [11]. Recent articles demonstrate that two gene sets of dysregulated genes could predict pCRT response in patients with rectal adenocarcinoma, one gene set is AKR1C3, CXCL11, CXCL10, IDO1, CXCL9, MMP12, HLA-DRA and another gene set is TMEM188, ITGA2, NRG, TRAM1, BCL2L13, MYO1B, KLF7,and GTSE1 [12, 13]. microRNAs (miRNAs) are small non-coding RNAs, which negatively regulates mRNA expression of targeted genes. In rectal cancer, miR-92a expression is inversely associated with KLF4 and IQGAP2 expression [14]. miR-573,miR-579 and miR-802 display the significant correlation with overall survival of patients, in addition to, miR-573 is significantly correlated with tumor grade of patients after preoperative chemoradiotherapy [15]. In patients with rectal adenocarcinoma, serum level of miR-125b is significantly over-expressed in pCRT non-responders compared to those responders [16].

In order to explore the tumorigenesis mechanism and potential biomarkers for early diagnosis of rectal adenoacrcinoma, integrated analyses of miRNA expression profiling, mRNA expression profiling and DNA methylation data based on The Cancer Genome Atlas (TCGA) database were performed. Our study might be the ground work for further mechanism elucidation of rectal adenocarcinoma and identification of the diagnostic biomarkers for early stage of rectal adenocarcinoma.

## Materials and methods

### Source of data

The mRNA expression data, miRNA expression data, methylation data and the corresponding clinical data for retal adenocarcinoma (READ) were downloaded from TCGA database (https://tcga-data.nci.nih.gov/tcga/, Oct 20, 2015). Total of 16 READ patients were excluded from 171 READ patients based on the criteria including patients without the history of other malignancy and without the history of neoadjuvant treatment before collection of tumor specimens. The sequencing platforms of mRNA expression data, miRNA expression data, methylation data were respective UNC_ IlluminaHiseq_RNASeqV2, BCGSC_IlluminaHiSeq_miRNASeq and JHU_USC_HumanMethylation450. There were respective 29 READ patients with TNM sage I, 49 patients with TNM sage II, 48 patients with TNM sage III, 23 patients with TNM sage IV and 6 patients with unknown TNM stage in our study.

### Identification of differentially expressed mRNAs and miRNAs

The mRNA expression profiling and miRNA expression profiling of READ and normal control tissues were downloaded from TCGA data portal. The mRNA and miRNA expression level were calculated and demonstrated as reads per million miRNA mapped data. The significantly differentially expressed mRNAs (DEmRNAs) and differentially expressed miRNAs (DEmiRNA) were identified inREAD compared to normal control tissues through DESeq2 repackage [17]. The calculated raw *P*-value was performed to multiple testing corrections through Benjamini and Hochberg method [18]. The adjusted *P*-value was described as false discovery rate (FDR). mRNA and miRNA with FDR<0.0001 and abs (log_2_ fold change)>2 was identified as DEmRNA and DEmiRNA, respectively.

### Differentially methylated CpG sites analysis

COHCAP package in R was applied to identify differentially methylated sites between READ and normal control samples [19]. *t*-test was used to determine differences in two group comparison. The methylation score for each CpG site was described as a beta value according to the fluorescent intensity ratio. The differentially methylated sites with FDR <0.05 and abs (delta beta) >0.2 were selected. The corresponding genes and CpG islands of the differentially methylated sites were annotated.

### Manhattan plot analysis and heatmap analysis

The distribution of differentially methylated CpG sites in 22 chromosomes was described as Manhattan plot by “qqman” package in R language. The pattern of differentially methylated CpG sites in READ and normal controls were visualized by “pheatmap” package in R language.

### DEmiRNA target genes prediction analysis

The target DEmRNAs of DEmiRNAs were predicted through mirWalk database [20], in which the targeted correlations between miRNAs and mRNAs have been confirmed through *in vivo* and *in vitro* experiments. 6 algorithms including RNA22, miRanda, miRDB, miRWalk, PICTAR2 and Targetscan were conducted to predict target-genes of miRNAs. The genes, simultaneously predicted by more than 4 algorithms, were identified as the target-genes of miRNAs [20].

### Visualization of DEmiRNA-DEmRNA network

The identified negative correlations between DEmiRNAs and DEmRNAs through PCC analysis and mirWalk database were visualized by Cytoscape software (http://cytoscape.org) [21]. In the network, a circular node represented the miRNA and a rectangle node represented the mRNA, and their association was represented by the solid line.

### Correlation analysis of aberrant gene expression and DNA methylation in READ

The identified DEmRNAs and reference genes with aberrantly methylated CpG islands were screened out to analyze the correlation between gene expression and DNA methylation. There was a negative correlation between DNA methylation and mRNA expression. The genes with hypermethylation and lower expression or genes with hypomethylation and higher expression in READ were our concern.

### KEGG pathway enrichment analysis

In order to explore the enriched signaling pathways of aberrantly methylated genes and aberrantly expressed genes, the online software of KOBAS 2.0 was used for Kyoto Encyclopedia of Genes and Genomes (KEGG) pathway enrichment [22, 23]. KOBAS 2.0 was updated on January 26th, 2015, contains the latest data of signaling pathways. *P*<0.05 was set as the threshold of significantly enriched pathways.

### Quantitative Real-Time Polymerase Chain Reaction (qRT-PCR)

5 paired READ and adjacent normal tissues were obtained from patients underwent radical surgery in Department of Colorectal Surgery, Tianjin Union Medical Center. Our study was approved by the Ethics committee of Tianjin Union Medical Center and complies with the Declaration of Helsinki. All of participants provided their written informed consent to participate in this study. Total RNA of fresh samples of 5 paired READ tumor and adjacent non-tumor tissues and was extracted by using TRIzol reagent (Invitrogen, CA, USA). The SuperScript III Reverse Transcription Kit (Invitrogen, CA, USA) was used to synthesize the cDNA.qRT-PCR reactions were carried out using Power SYBR Green PCR Master Mix (Applied Biosystems, Foster City, CA) on the Applied Biosystems 7500 (Applied Biosystems, Foster City, CA).The relative expression of genes was calculated using the 2^-ΔΔct^ methods [24].GAPDH was used as internal control gene and all of primers used were shown in Table 1. Each assay was performed as in triplicate. The GraphPad Prism version 6.0 software packages (GraphPad Software, San Diego, CA, USA) was used to output the Figures.

**Table 1:** The primers for qRT-PCR validation.

Genes	Primers
ATP2B4	Forward-CGTGGTGTTAGTGACTGCCT
	Reverse-GGGAGCTGGATGAGTTGACC
NR3C1	Forward-ACTGCCCCAAGTGAAAACAGA
	Reverse-ATGAACAGAAATGGCAGACAT
PRKCB	Forward-CTTCAAGCAGCCCACCTTCT
	Reverse-TTCCCGAAGCCCCAGATG
HAPLN1	Forward-CTGAAGGGAGGCAGTGATAG
	Reverse-ACCACACCTTGTAAGTCCAGT
ROR1	Forward-CAGTCAGTGCTGAATTAGTGCC
	Reverse-TCATCGAGGGTCAGGTAAGAAT
TCF7	Forward-TTGATGCTAGGTTCTGGTGTACC
	Reverse-CCTTGGACTCTGCTTGTGTC
WNT2	Forward-GCCCCTCTCGGTGGAATCTGGCTC
	Reverse-GCTGTGATCCCTGTCCAGGGTGTT
ONECUT2	Forward-AGGCTGCCTACACCGCCTATC
	Reverse-GCCGTCCAGGATCGAGGCCAT
PDPN	Forward-GGAAGGTGTCAGCTCTGCTC
	Reverse-CGCCTTCCAAACCTGTAGTC

qRT-PCR: quantitative real-time polymerase chain reaction.

### The expression levels of DEmRNAs were validated based on Gene Expression Omnibus database

Gene Expression Omnibus (GEO, https://www.ncbi.nlm.nih.gov/geo/) is a freely public data repository that archives microarray, next-generation sequencing, and other forms of high-throughput functional genomics data submitted by research community. In order to performed the further validation of dysregulated genes in READ, public expression profiling deposited in GEO database was used in our study.

Firstly, RNA-seq expression profiling of READ tissues were searched, however, none of dataset was retrieved. Then, microarray expression profiling of READ tissues were searched and the inclusion criteria of datasets were complied with following details: (1) the dataset was generated from mRNA expression profiling of READ patients; (2) both of READ and matched mucosa tissues samples were available in the dataset; (3) the sample size of dataset was more than 50. Finally, GSE75970 (230 READ versus 230 matched mucosa tissues) and GSE20842 (65 READ versus 65 matched mucosa tissues) of mRNA expression profiling were incorporated into our study.

The mRNA expression profiling of GSE75970 and GSE20842 were integrated and the expression levels of f DEmRNAs in READ were detected. Box-plot analysis was performed to describe the expression of DEmRNAs both in READ and matched mucosa tissues. *P*-value indicating the difference between two group was calculated and *P*<0.05 was significant difference.

## Results

### Dysregulated mRNAs in READ

The mRNA expression profiling of READ tumor samples and normal control tissues were conducted to differentially expressed genes analysis based on TCGA database (Table 2). Total of 1192 DEmRNAs including 439 up-regulated and 753 down-regulated DEmRNAs were identified in READ compared to normal controls according to the threshold of FDR<0.001 and abs (log_2_ fold change)>2. KRT23, FOXQ1 and DPEP1 were the top 3 significantly up-regulated DEmRNAs; DPP6, PLP1 and SCN7A were the top 3 significantly down-regulated DEmRNAs in READ, as Table 3 shown.

**Table 2:** The basic information of READ datasets.

Data Type	Platform	case	control
Methylation	Methyl JHU-USC HumanMethylation450	93	7
mRNA	UNC IlluminaHiseq_RNASeqV2	89	10
miRNA	miRNASeq BCGSC IlluminaHiSeq-miRNASeq	88	3

READ: rectal adenocarcinoma.

**Table 3:** The top 10 dysregulated mRNAs in READ.

Gene symbol	Gene ID	log_2_FC	P-value	FDR
**Up-regulation**				
KRT23	25984	6.931839	1.17E-35	7.94E-33
FOXQ1	94234	6.699067	3.29E-71	1.34E-67
DPEP1	1800	6.694418	3.38E-46	5.52E-43
COL10A1	1300	6.344651	7.60E-27	1.70E-24
KRT80	144501	6.169529	1.83E-77	1.50E-73
MMP7	4316	6.1289	2.59E-30	9.83E-28
COL11A1	1301	6.060277	1.22E-29	4.00E-27
LOC100190940	100190940	6.031999	4.53E-32	2.31E-29
NOTUM	147111	5.878586	8.84E-21	8.44E-19
STRA6	64220	5.876284	2.38E-31	1.05E-28
**Down-regulation**				
DPP6	1804	-5.73634	9.57E-27	2.08E-24
PLP1	5354	-5.60733	4.52E-21	4.61E-19
SCN7A	6332	-5.41832	5.82E-20	4.87E-18
BEST4	266675	-5.38243	5.44E-30	1.85E-27
NRXN1	9378	-5.29459	4.71E-21	4.78E-19
NBLA00301	79804	-5.29166	1.26E-22	1.73E-20
CA7	766	-5.12203	1.82E-18	1.18E-16
NPTX1	4884	-5.05257	2.10E-21	2.38E-19
HRNBP3	146713	-4.96212	4.68E-24	7.89E-22
RSPO2	340419	-4.94184	3.78E-21	3.97E-19

FC: fold change; FDR: false discovery rate; READ: rectal adenocarcinoma.

### Dysregulated miRNAs in READ

27 miRNAs with aberrant expression were screened in READ, which covered 17 up-regulated and 10 down-regulated DEmiRNAs in READ according to the screening criteria of FDR<0.001 and abs (log_2_ fold change)>5. As Table 4 shown, hsa-mir-424, hsa-mir-215 and hsa-mir-374a were the top 3 significantly up-regulated DEmiRNAs; hsa-mir-328, hsa-mir-129-1 and hsa-mir-129-2 were the top 3 significantly down-regulated DEmiRNAs in READ compared to normal tissues.

**Table 4:** All of dysregulated miRNAs in READ.

miRNA	log_2_FC	FDR
**Up-regulation**		
hsa-mir-424	7.307456105	1.50E-12
hsa-mir-215	6.581769019	2.36E-10
hsa-mir-374a	6.532649212	3.47E-30
hsa-mir-19b-2	6.166244957	3.48E-18
hsa-mir-144	6.127227998	8.53E-10
hsa-mir-452	5.997431458	6.48E-17
hsa-let-7f-2	5.970012196	5.47E-35
hsa-mir-20a	5.909272735	4.44E-32
hsa-mir-21	5.867000542	9.06E-78
hsa-mir-142	5.803640777	8.24E-30
hsa-mir-335	5.748131674	5.98E-22
hsa-mir-1-2	5.407168809	2.27E-07
hsa-mir-542	5.245107289	6.37E-16
hsa-mir-429	5.237487024	4.51E-20
hsa-mir-141	5.084327165	6.98E-33
hsa-mir-101-2	5.073334552	8.53E-10
hsa-mir-182	5.058384389	1.30E-17
**Down-regulation**		
hsa-mir-328	-6.996143139	3.21E-48
hsa-mir-129-1	-6.114541173	4.10E-15
hsa-mir-129-2	-6.02843377	1.10E-17
hsa-mir-139	-5.893124606	3.19E-29
hsa-mir-149	-5.844766545	8.89E-29
hsa-mir-766	-5.536535274	1.34E-40
hsa-mir-197	-5.28419299	1.15E-49
hsa-mir-486	-5.231756891	3.20E-14
hsa-mir-574	-5.165489306	5.51E-30
hsa-mir-1249	-5.083479596	2.59E-22

FC: fold change; FDR: false discovery rate; READ: rectal adenocarcinoma.

### Aberrant methylation CpG sites in READ

**Fig 1:**
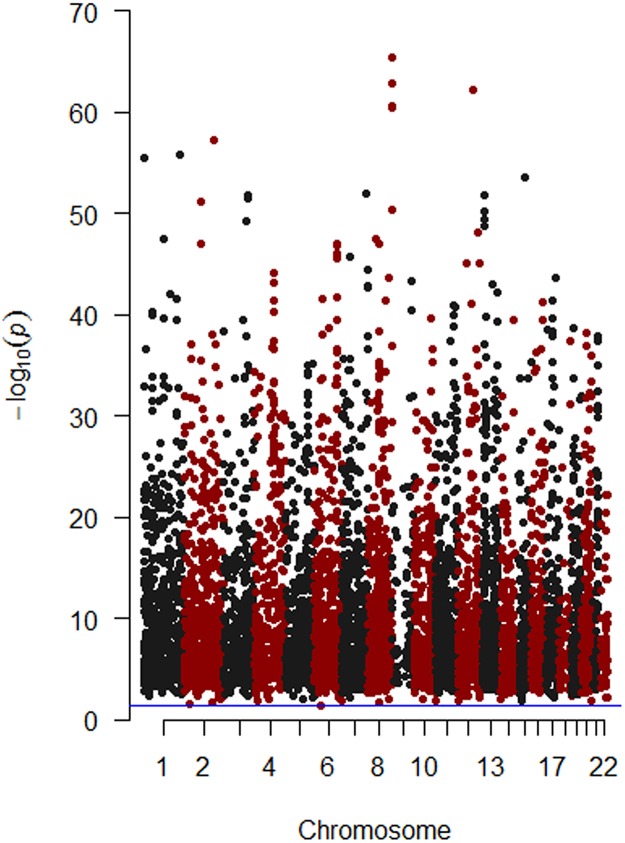
Manhattan plot of the differentially methylated CpG sites for READ. The red dots indicated differentially methylated CpG sites in READ locating on chromosome 2, 4, 6, 8, 10, 12, 14, 16, 18, 20 and 22; the black dots indicated differentially methylated CpG sites in READ locating on chromosome 1, 3, 5, 7, 9, 11, 13, 15, 17, 19 and 21. The purple-horizontal solid line is the significant level (*P*-value<0.05) for identification of differentially methylated CpG sites.

**Fig 2:**
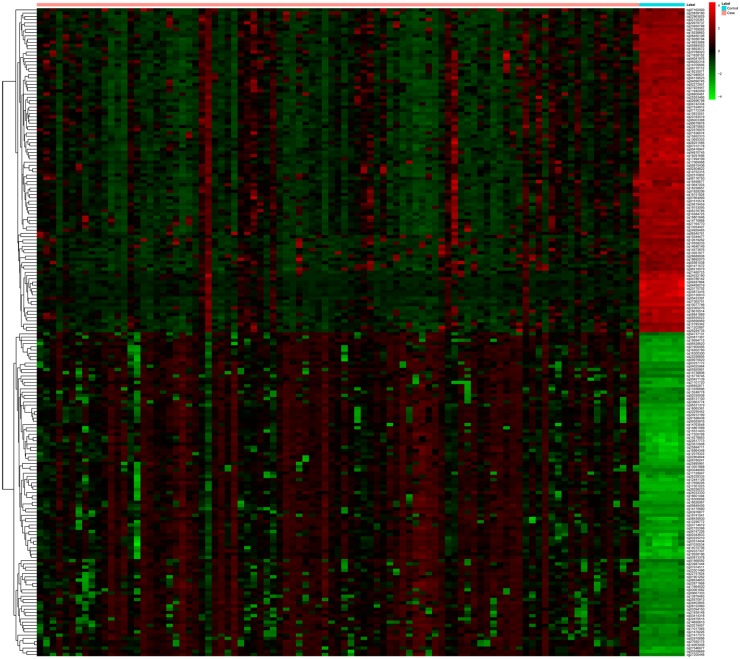
Heat map visualization of the patterns of methylation change for the top 100 hypermethylation CpG sites and top 100 hypomethylation CpG sites in READ compared to normal control tissues. Red and green dot respectively indicated hypermethylation and hypomethylation.

In order to investigate the differentially methylated sites between READ and normal control samples, methylation score of each gene, described as a beta value, was compared between READ and normal controls. Finally, 6403 differentially methylated sites including 4275 hypermethylated sites and 2128 hypomethylated sites were identified in READ (S1 Table). Fig 1 showed the distribution of differentially methylated CpG sites in 22 chromosomes. The top 100 hypermethylation and top 100 hypomethytion CpG sites in READ were subjected to heatmap analysis. The difference of CpG sites pattern between READ and normal control tissues was notable, as Fig 2 shown. In addition, 6403 differentially methylated sites were located in 555 hypermethylated and 6 hypomethylation CpG islands, associated with 446 annotated genes (S2 Table).

### DEmiRNA-DEmRNA interaction in READ

In order to explore the interaction between DEmiRNA and DEmRNA, DEmiRNA was subjected to mirWalk database, and then the predicted target-genes of DEmiRNAs in READ were obtained. Total of 668 DEmRNAs were identified as the target-genes of 27 DEmiRNAs in READ.

The DEmiRNA-mRNA interaction network was visualized by Cytoscape software. As S1 Fig shown, there are 695 nodes and 1987 edges in the network. hsa-mir-335, hsa-mir-20a, hsa-mir-19b-2, hsa-mir-452 and hsa-mir-182 had the highest connectivity with genes, regulated 254, 131, 122, 113 and 108 DEmRNAs, respectively, while LONRF2, PGR, KIAA2022, PDE4D and ZDHHC15 were respectively regulated by 14, 12, 12, 11 and 11 DEmiRNAs.

**Fig 3:**
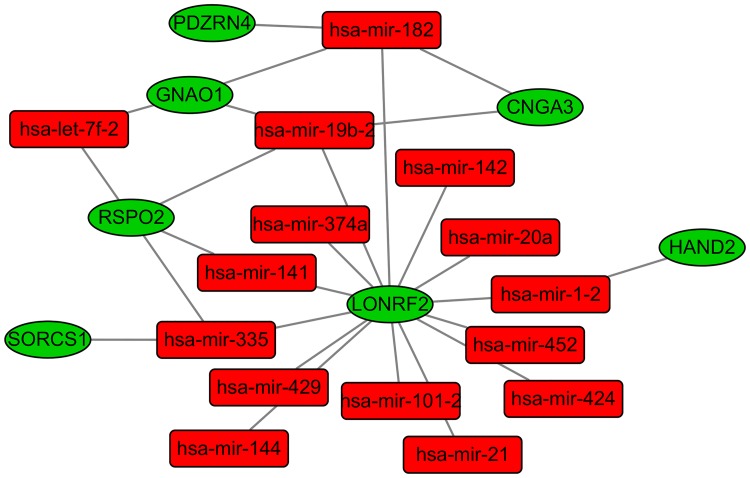
The sub-network among 7 DEmRNAs and DEmiRNAs in READ. Rectangle node represented DEmiRNA and circular node represented DEmRNA. The red and green colors represent up-regulation and down-regulation in READ, respectively.

As Fig 3 revealing, the sub-network among 7 DEmRNAs which were our concerns and DEmiRNAs in READ deciphered that LONRF2 was negatively regulated by hsa-mir-335, hsa-mir-20a and hsa-mir-452; has-mir-182 negatively correlated with PDZRN4, GNAO1 and CNGA3; and hsa-mir-19b-2 negatively regulated GNAO1 and CNGA3.

### Correlation analysis of aberrant gene expression and DNA methylation in READ

In order to determine the potential effects of the aberrant DNA methylation on the abnormal gene expression in READ, the association between DNA methylated status and mRNA expression level were investigated. DNA methylation status could positively or negatively correlate with mRNA expression profiling. In our study, negative correlation between DNA methylation and gene expression in READ were concerned.

**Fig 4:**
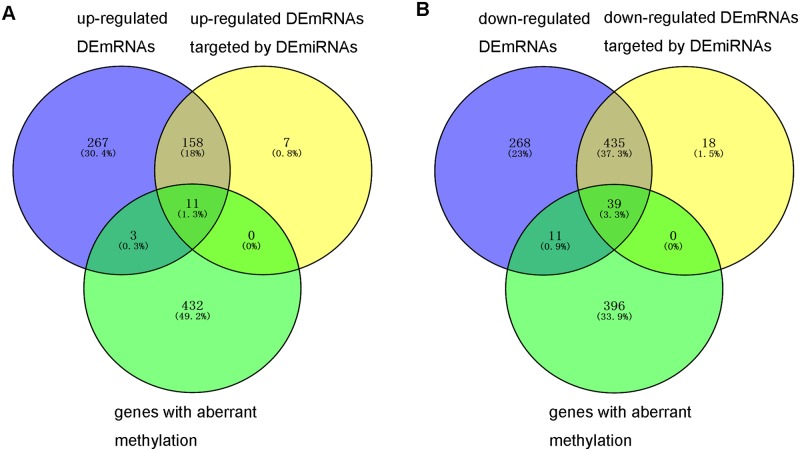
The comparison of DEmRNAs, DEmRNAs targeted by DEmiRNAs and differentially methylated genes. (A) comparison of up-regulated DEmRNAs, up-regulated DEmRNAs targeted by DEmiRNAs and differentially methylated genes. (B) comparison of down-regulated DEmRNAs, down-regulated DEmRNAs targeted by DEmiRNAs and differentially methylated genes.

555 hypermethylated CpG islands were associated with 441 annotated genes and 6 hypormethylated CpG islands were associated with 5 annotated genes, respectively. 446 genes with aberrant methylation CpG islands were overlapped with 668 genes with aberrant expression, total 50 dysregulated genes including 11 up-regulated genes and 39 down-regulated genes with hypeymethylated CpG islands were identified, and there was no dysregulated genes with hypomethylated CpG sites were identified in READ as Fig 4 shown (S3 Table).

### Functional annotation

In order to investigate the potential signaling pathways of 50 dysregulated gene with aberrant methylation in READ, KEGG database was used to enrich the pathways. As Table 5 shown, total of 9 pathways including cAMP signaling pathway, circadian entrainment, glutamatergic synapse, nicotine addiction, amphetamine addiction, morphine addiction, melanogenesis, retrograde endocannabinoid signaling and cholinergic synapse was significantly enriched (*P*< 0.05). GRIA4, GNAO1 and GRIN2D were enriched in 6, 6 and 5 pathways.

**Table 5:** The pathway enrichment of 50 dysregulated genes with abnormal methylation.

KEGG Term	KEGG ID	*P*-Value	Genes
cAMP signaling pathway	hsa04024	0.0004	VIPR2,CNGA3,PDE4D,GRIN2D,GRIA4
Circadian entrainment	hsa04713	0.0036	GNAO1,GRIA4,GRIN2D
Glutamatergic synapse	hsa04724	0.0058	GNAO1,GRIA4,GRIN2D
Nicotine addiction	hsa05033	0.0076	GRIA4,GRIN2D
Amphetamine addiction	hsa05031	0.0196	GRIA4,GRIN2D
Morphine addiction	hsa05032	0.034	PDE4D,GNAO1
Melanogenesis	hsa04916	0.0403	WNT2,GNAO1
Retrograde endocannabinoid signaling	hsa04723	0.041	GNAO1,GRIA4
Cholinergic synapse	hsa04725	0.0484	KCNQ5,GNAO1

### qRT-PCR verification

**Fig 5:**
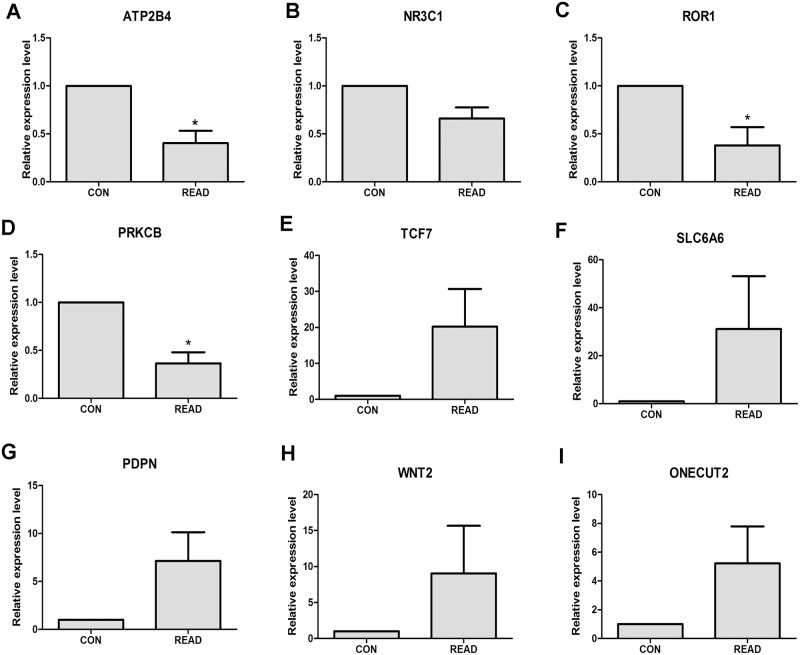
The verification of the expression level of DEmRNAs in 5 READ samples and 5 adjacent tumor tissues through qRT-PCR. READ represented rectal adenocarcinoma, and CON represented adjacent tumor tissues. * was *P*<0.05. (A) ATP2B4; (B); NR3C1 (C); ROR1(D); PRKCB; (E) TCF7;(F) SLC6AC;(G) PDPN;(H) WNT2;(I) ONECUT2.

In our study, dysregulated genes were identified in READ through bioinformatics analysis. qRT-PCR method was used to validate the expression levels of dysregulated genes in 5 READ tumor samples and 5 adjacent non-tumor tissues. As Fig 5A, 5C and 5D shown, the expression levels of ATP2B4 (*P*<0.05), ROR1 (*P*<0.05) and PRKCB (*P*<0.05) were significantly down-regulated and NR3C1 (Fig 5B) was down-regulated in READ compared to adjacent non-tumor tissues. TCF7, SLC6A6, PDPN, WNT2 and ONECUT2 were up-regulated in READ, as Fig 5E–5I shown.

### The expression levels of DEmRNAs were analyzed in the GSE75970 and GSE20842 datasets

**Fig 6:**
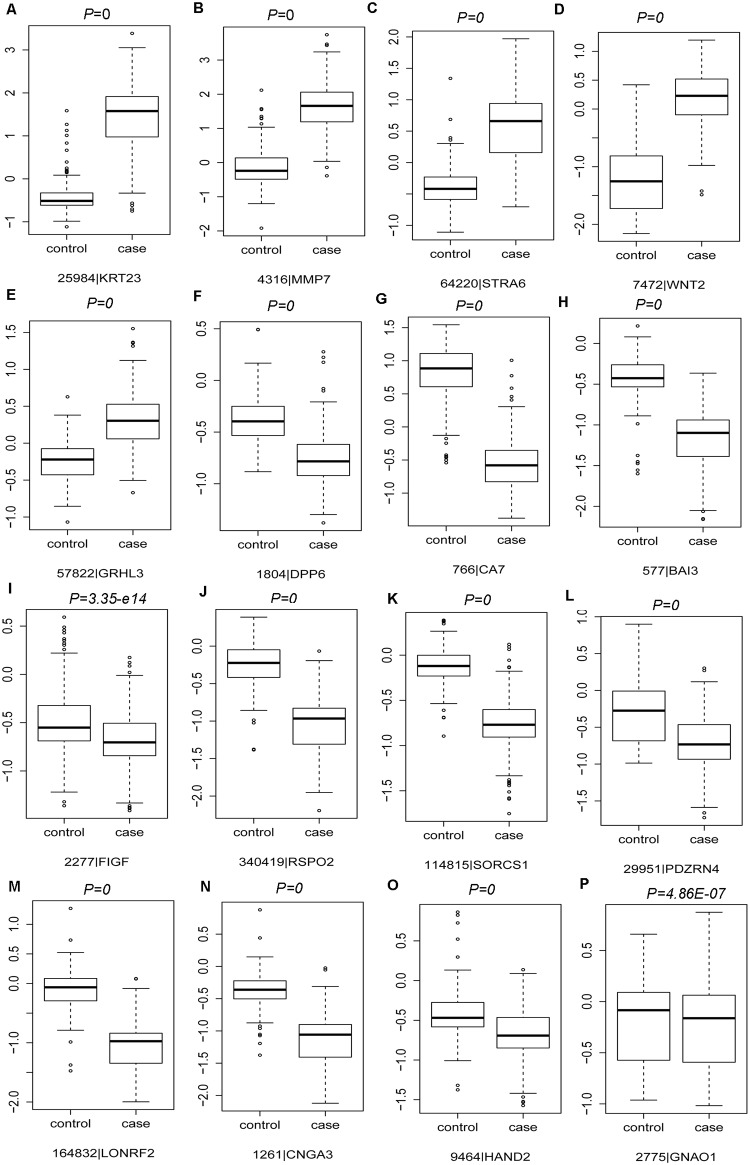
Box-plot indicated the expression levels of 16 candidate DEmRNAs of READ tissues compared with matched mucosa tissues in the GSE75970 and GSE20842 datasets. (A):KRT23; (B):MMP7; (C):STRA6; (D):WNT2; (E):GRHL3; (F):DPP6; (G):CA7; (H):BAI3;(I):FIGF; (J):RSPO2; (K):SORCS1; (L):PDARN4; (M):LONRF2; (N):CNGA3; (O):HAND2;(P):GNAO1.

Integrated analyses were performed to GSE75970 and GSE20842 datasets for detecting the expression levels of identified DEmRNAs in READ through the larger sample size. In our study, 33 DEmRNAs’ expression were detected in GSE75970 and GSE20842 datasets, including 10 DEmRNAs randomly selected from top 20 up-regulated DEmRNAs, 10 DEmRNAs randomly selected from top 20 down-regulated DEmRNAs, 9 DEmRNAs examined by qRT-PCR and 7 DEmRNAs discussed in the manuscript (Fig 6 and S2 Fig).

As Fig 6A–6E revealed, the expression levels of KRT23, MMP7, STRA6, WNT2, GRHL3 were significantly up-regulated in READ tissues; moreover, the expression levels of DPP6, CA7, BAI3, FIGF, RSPO2, SORCS1, PDZRN4, LONRF2, CNGA3, HAND2 and GNAO1 were obviously down-regulated in READ tissues compared with matched mucosa tissues based on the microarray analyses (Fig 6F–6P). The expression levels of other 17 DEmRNAs in READ tissues through microarray analyses were shown in S2 Fig. In summary, the expression levels of those 33 DEmRNAs in READ tissues based on microarray analyses were completely compatible with our bioinformatics analyses based on TCGA datasets.

## Discussion

In our study, DEmiRNAs, DEmRNAs and differentially methylated genes were identified in READ compared with normal control tissues based on the TCGA database. DEmiRNA-DEmRNA regulatory network was constructed and DEmRNAs associated with differentially methylated genes were recognized. qRT-PCR was applied to verify the dysregulated genes through bioinformatics analysis. 9 candidate genes were randomly selected for qRT-PCR examination, ATP2B4, NR3C1, ROR1 and PPKCB were down-regulated in READ, TCF7, SLC6A6, PDPN, WNT2 and ONECUT2 were up-regulated in READ compared to paired adjacent non-tumor tissues, which were accordance with our analyses. In order to further validate the expression levels of DEmRNAs in READ, microarray expression profiling with larger sample size of READ and matched mucosa tissues generated from GEO database were applied for validation. The expression status of 33 representative DEmRNAs in READ tissues based on microarray analyses were completely compatible with our bioinformatics analyses based on TCGA datasets. Both of qRT-PCR validation and microarray analyses indicated our bioinformatics analyses based on TCGA datasets was acceptable.

7 genes SORCS1, PDZRN4, LONRF2, CNGA3, HAND2, RSPO2 and GNAO1 were our concerns by reasons of those down-regulated genes had increased methylation and were negatively targeted by up-regulated miRNAs in READ.

*RSPO2* encodes R-spondin 2, belongs to R-spondin family. RSPO2, is a typical secretory protein and activates the canonical Wnt/beta-catenin signaling pathway as an agonist. It is negatively regulated by mir-335, mir-141, mir-19b-2 and let-7f-2 in the DEmiRNA-DEmRNA network (Fig 3). RSPO2 with hypermethylation was the top 10 down-regulated DEmRNAs in READ compared to normal control tissues. Dysregulated expression of RSPO2 is closely related to the tumor invasiveness and aggressiveness in papillary thyroid cancer, pancreatic cancer and lung adenocarcinoma [25–27]. However, RSPO2 functions as a tumor driver or suppressor in CRC is still controversial [28–30]. Our study indicated RSPO2 was significantly down-regulated in READ and kept the line with the published research, which demonstrates RSPO2 functions as a tumor suppressor; over-expression of it inhibits CRC cell proliferation and tumorigenicity [28]. RSPO2 is highly expressed in colon cancer stem cells and promotes the invasion of CRC cells through enhancing epithelial-mesenchymal transition [26]. The biological functions of RSPO2 in CRC and READ are unclear and the expression pattern of RSPO2 in READ regulated by DNA methylation modification and miRNA needs to be further investigated through experiments.

*GNAO1* and *PDZRN4* with increased methylation were down-regulated in READ. *GNAO1* encodes the guanine nucleotide-binding protein, α-activating activity polypeptide O, is a member of the subunit family of Gα proteins. GNAO1 with hypermethylation was down-regulated in READ, and was negatively targeted by mir-182, mir-19b-2 and let-7f-2 (Fig 3). GNAO1 was significantly enriched in circadian entrainment, glutamatergic synapse, morphine addiction, melanogenesis, retrograde endocannabinoid signaling and cholinergic synapse pathways. A number of articles have illuminated its biological roles in cancers including hepatocellular carcinoma (HCC), gastric cancer and renal cell carcinoma [31–33].In HCC, GNAO1 acts as tumor suppressor depending on down-regulation of GNAO1 promoting cell proliferation and suppressing the senescence of HCC cells [31]. Nevertheless, GNAO1 functions as tumor driver in gastric cancer. The silencing of GNAO1 in gastric cancer cells inhibits cell proliferation and enhances cell apoptosis; increased GNAO1 predicts the unfavorable prognosis in patients with gastric cancer [32]. PDZRN4 encodes PDZ domain containing ring finger 4, PDZRN4 was negatively targeted by mir-182 (Fig 3). PDZRN4 is described as a potential tumor suppressor with down-regulation in HCC. Increased expression of it inhibits cell proliferation and colony formation [34].

*HAND2* encodes heart and neural crest derivatives expressed 2, belongs to the basic helix-loop-helix family of transcription factors. HAND2 was negatively regulated by mir-1-2 (Fig 3), which is down-regulated in READ with increased methylation. It is related to endometrial carcinoma, non-small cell lung cancer and neuroblastoma [35–37]. HAND2 is absent in endometrioid carcinoma and reduced in atypical hyperplasia compared to benign endometrium; knockout of HAND2 leads to continuous proliferation in mice model [35]. HAND2, as the transcript factor, is over-expressed in early stage of lung squamous cell carcinoma instead of lung adenocarcinoma [36]. HAND2 and DEIN expression is well correlated in neuroblastoma, HAND2 is highly expressed in neuroblastoma [37].

SORCS1 and CNGA3 with increased methylation were down-regulated in READ. In our work, SORCS1 were negatively regulated by mir-335 and CNGA3 was negatively regulated by mir-182 and mir-19b-2 (Fig 3). *SORCS1* encodes sortilin related VPS10 domain containing receptor 1,is associated with the Alzheimer's disease, type 2 diabetes and obese women with polycystic ovary syndrome[38–40]. CNGA3 encodes cyclic nucleotide gated channel alpha 3, a member of cyclic nucleotide-gated cation channel protein family, is required for normal vision. The missense mutations for c.633T>A (p.D211E) and c.1006G>T (p.V336F) and the combined heterozygous mutations for c.997_998delGA and p.M424V in the CNGA3 gene is the cause for achromatopsia [41, 42].

*LONRF2*, locates at chromosome 2, encodes LON peptidase N-terminal domain and ring finger 2. The gene is conserved in various species covered chimpanzee, mice dog, chicken, zebrafish and frog. In our study, LONRF2 was one of significantly down-regulated DEmRNAs, and was negatively targeted by 14 miRNAs, such as mir-21, mir-1-2, mir-335, mir-141, mir-182, mir-19b-2, mir-20a, mir-101-2, mir-142, mir-429, mir-144, mir-374a, mir-452 and mir-424 (Fig 3). As far as we know, there is few published research reports LONRF2 associated with disease; our study firstly reported that LONRF2 with hypermethylation was identified as the novel DEmRNA in READ.

In conclusion, DEmRNAs, DEmiRNAs and differentially methylated regions were identified in READ compared to normal control tissues based on TCGA database. 39 down-regulated DEmRNAs with hypermethylation, had potentially negative correlation with DEmiRNAs, were identified in READ. Among these 39 genes, RSPO2, GNAO1, PDZRN4, HAND2, SORCS1, CNGA3 and LONRF2 might play essential roles in the tumorigenesis of READ. GNAO1, PDZRN4, HAND2, SORCS1, CNGA3 and LONRF2 are not previously described in relation to tumorigenesis of READ. The role of RSPO2 in colorectal cancer is arguable according to the published article. Our results provide the foundation for further READ research in mechanism elucidation and early diagnosis, and these results must be confirmed through additional experiments.

## Supporting information

S1 Fig DEmiRNA-DEmRNA regulatory network in READ. Rectangle node represented DEmiRNA and circular node representsed DEmRNA. The red and green colors represented up-regulation and down-regulation in READ, respectively. (A) The network of up-regulated DEmiRNA interaction with down-regulated DEmRNAs in READ. (B) The network of down-regulated DEmiRNA interaction with up-regulated DEmRNAs in READ.
(TIF)

S2 Fig Box-plot indicated the expression levels of other 17 candidate DEmRNAs in READ tissues compared with matched mucosa tissues in the GSE75970 and GSE20842 datasets. (A):FOXQ1; (B):COL11A1; (C):NOTUM; (D):GRIN2D2; (E):COMP; (F):BEST4; (G):NBLA00301; (H):ATP1A2; (I):MT1M; (J):AQP8; (K):ATP2B4; (L):NR3C1; (M):ROR1; (N): PPKCB; (O):SLC6A6; (P):PDPN; (Q): ONECUT2.
(TIF)

S1 Table Total of 6403 differentially methylated sites were identified in READ. (XLSX)

S2 Table 6403 differentially methylated sites were located in 561 aberrant methylation CpG islands, associated with 446 annotated genes. (XLSX)

S3 Table 50 DEmRNAs with dysregulated expression and aberrant methylation CpG islands were identified in READ. (XLSX)
