# Supplementary figures and images for: Abnormal expression of mRNA, microRNA alteration and aberrant DNA methylation patterns in rectal adenocarcinoma
Source: PLoS One. 2017 Mar 28;12(3):e0174461. doi: 10.1371/journal.pone.0174461 (PMC5370119; doi:10.1371/journal.pone.0174461)

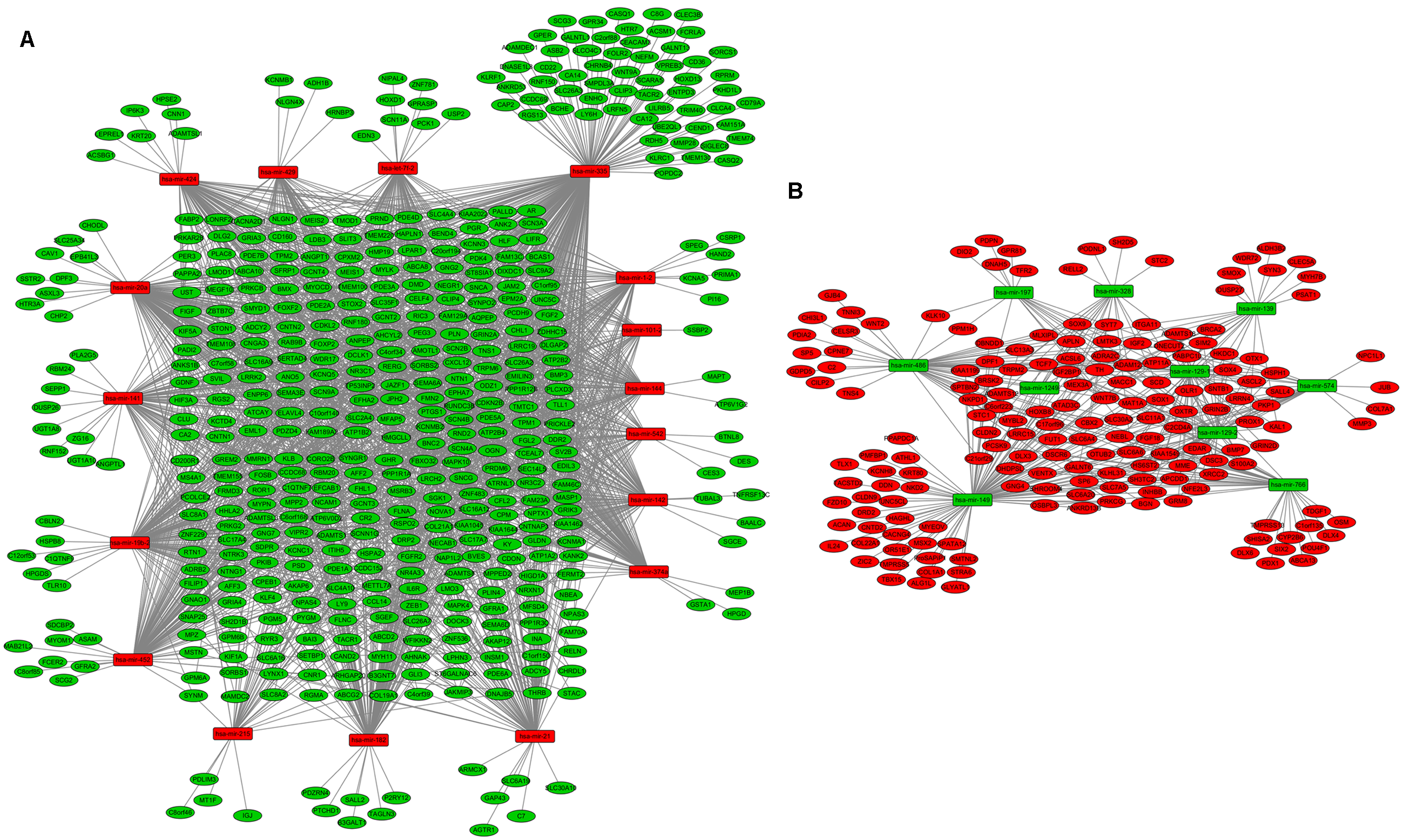

Supplement: S1 Fig — Rectangle node represented DEmiRNA and circular node representsed DEmRNA. The red and green colors represented up-regulation and down-regulation in READ, respectively. (A) The network of up-regulated DEmiRNA interaction with down-regulated DEmRNAs in READ. (B) The network of down-regulated DEmiRNA interaction with up-regulated DEmRNAs in READ. (TIF) [file pone.0174461.s001.tif]

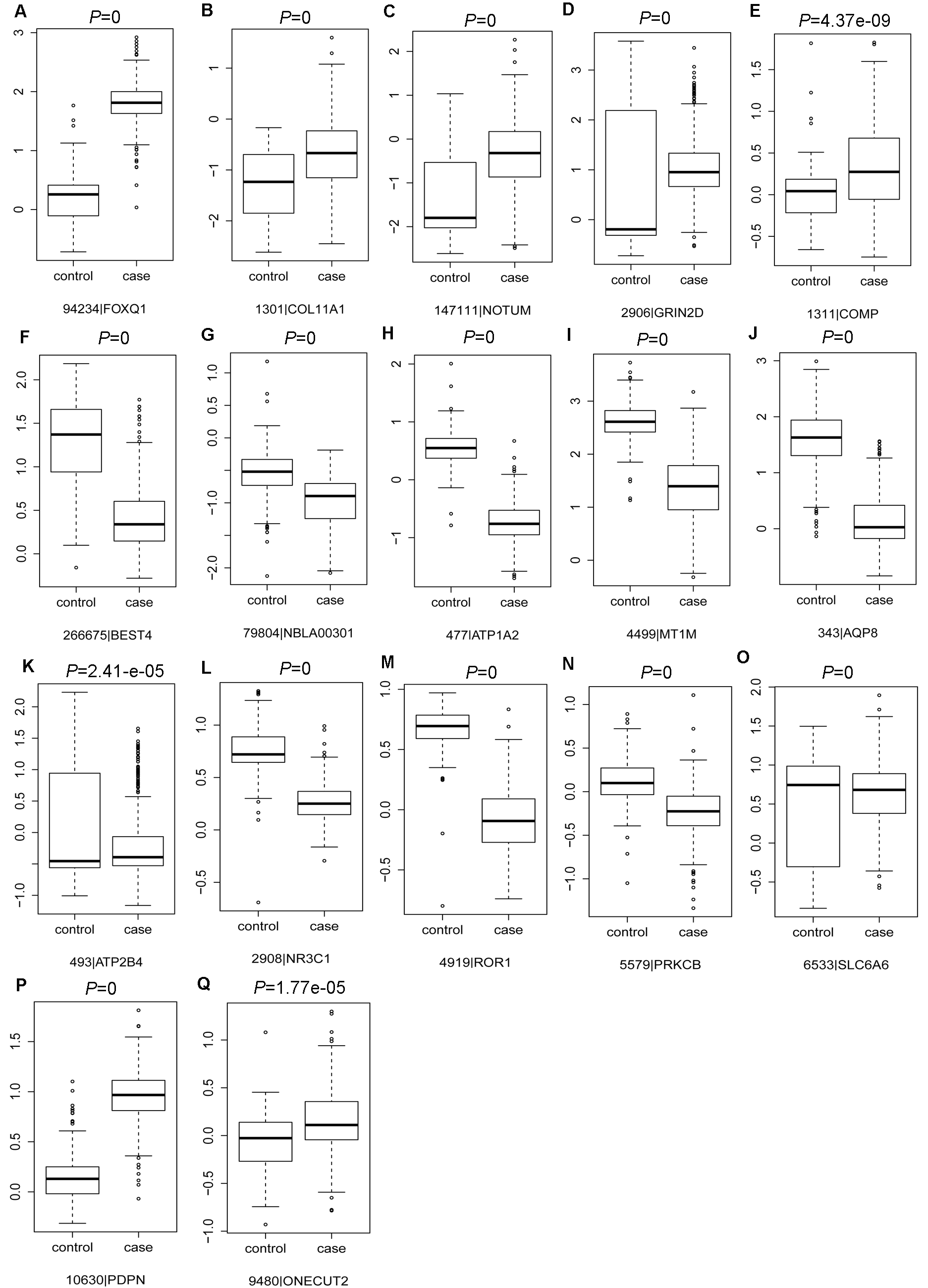

Supplement: S2 Fig — (A):FOXQ1; (B):COL11A1; (C):NOTUM; (D):GRIN2D2; (E):COMP; (F):BEST4; (G):NBLA00301; (H):ATP1A2; (I):MT1M; (J):AQP8; (K):ATP2B4; (L):NR3C1; (M):ROR1; (N): PPKCB; (O):SLC6A6; (P):PDPN; (Q): ONECUT2. (TIF) [file pone.0174461.s002.tif]
